# Non-Pharmacological Pulmonary Rehabilitation in Patients with Pneumoconiosis: A Systematic Review

**DOI:** 10.3390/arm94020022

**Published:** 2026-03-31

**Authors:** Madina B. Baurzhan, Sayagul A. Kairgeldina, Venera M. Almatova, Alexandr E. Gulyayev, Raushan S. Dosmagambetova, Kanat K. Tekebayev, Karashash Absatarova, Karlygash S. Absattarova

**Affiliations:** 1 Research Department, Research Institute of Balneology and Medical Rehabilitation, Ministry of Health of the Republic of Kazakhstan, Astana 010000, Kazakhstan; madina_baurzhan@mail.ru (M.B.B.); sanborovoe@mail.kz (S.A.K.); akin@mail.ru (A.E.G.); dosmagambetovaraushan@gmail.com (R.S.D.); kanat_7@mail.ru (K.K.T.); 2 Non-Profit Joint Stock Company “Astana Medical University”, Astana 010000, Kazakhstan; naite07@mail.ru; 3 Laboratory of Drug Discovery and Development, Nazarbayev University, Astana 010000, Kazakhstan; 4 Al-Farabi Kazakh National University, Almaty 050040, Kazakhstan; karashash.absatarova@kaznu.kz; 5 Department of Public Health, Non-Profit Joint Stock Company “Asfendiyarov Kazakh National Medical University”, Almaty 050000, Kazakhstan

**Keywords:** pneumoconiosis, occupational lung diseases, pulmonary rehabilitation, non-pharmacological therapy, quality of life, six-minute walk test, anxiety and depression, systematic review

## Abstract

**Highlights:**

**What are the main findings?**

- Pulmonary rehabilitation improves functional capacity and health-related quality of life in pneumoconiosis patients.
- Multicomponent rehabilitation programs provide more consistent physical and psychological benefits.

**What are the implications of the main findings?**

- Rehabilitation should be included in the long-term management of occupational dust-related lung diseases.
- Standardized functional tests and patient-reported outcomes improve the evaluation of rehabilitation effectiveness.

**Abstract:**

Background: Pneumoconiosis remains a major occupational lung disease associated with progressive respiratory impairment, reduced functional capacity, and diminished quality of life. Non-pharmacological rehabilitation has been increasingly proposed as a supportive intervention; however, evidence regarding its effectiveness remains heterogeneous. Objective: This study aimed to systematically review and synthesize the available evidence on the effects of non-pharmacological rehabilitation interventions on functional capacity, quality of life, and psychological outcomes in patients with pneumoconiosis. Methods: A systematic literature search was conducted in major electronic databases and grey literature sources in accordance with PRISMA 2020 guidelines. Studies evaluating non-pharmacological rehabilitation interventions in adults with pneumoconiosis were eligible for inclusion. Outcomes of interest included functional capacity, health-related quality of life, and psychological well-being. Due to methodological heterogeneity across studies, a qualitative synthesis was performed. Results: Six studies met the predefined inclusion criteria and were included in the qualitative synthesis. The reviewed evidence suggests that structured rehabilitation interventions were associated with clinically meaningful improvements in functional capacity, particularly in structured rehabilitation programs, most consistently reflected by increases in six-minute walk distance exceeding established minimal clinically important differences in three studies. Improvements in health-related quality of life and selected psychological outcomes were also reported, although outcome measures and intervention protocols varied across studies. Significant improvements in exercise capacity, dyspnea severity, and health-related quality of life were reported. Conclusions: Non-pharmacological rehabilitation may provide clinically meaningful benefits for patients with pneumoconiosis, based on limited and heterogeneous evidence, particularly in terms of functional capacity and quality of life. Nevertheless, the current evidence base is limited by heterogeneity in study design and outcome reporting. Further high-quality, standardized trials are needed to strengthen the evidence and guide the clinical implementation of rehabilitation programs for occupational lung diseases.

## 1. Introduction

The issue of respiratory pathologies occupies a leading position in the structure of occupational morbidity in the Republic of Kazakhstan. According to official national statistics, approximately 224,000 workers are exposed to hazardous production factors, of which about 170,000–180,000 operate under conditions of excessive dust and gas concentrations [1]. The most unfavorable situation is observed in the mining industry: in the Ulytau region, the share of employees working in hazardous conditions reaches 40.3%, while in the Karaganda region, it stands at 26.5%. The geographical determinism of these pathologies is clearly evidenced by the cases in the Karaganda (55% of all cases in the Republic of Kazakhstan, primarily in the coal industry) and East Kazakhstan regions (34%, concentrated in the mining and metallurgical complex), stemming from the high density of industrial enterprises in these areas [1,2].

A retrospective analysis covering the last 30 years demonstrates an undulating trend in the detection of pneumoconiosis and dust-induced bronchitis. While the 1990s and early 2000s were characterized by a fragmentation of the occupational pathology service and latent statistical reporting, the past 5–7 years have seen a significant surge in registrations (a 2.5-fold increase in primary diagnoses) [3]. Within the structure of occupational pathology, diseases caused by industrial aerosols consistently maintain a leading position, accounting for 40.4% of the total number of occupational diseases in 2023–2024. According to national statistics and publicly available analytical reports, the registration of 136 new cases of pneumoconiosis in 2023, amidst a general 10% increase in pulmonary pathologies, highlights the importance of strengthening monitoring systems and occupational health interventions within the industrial sector [1,4]. Furthermore, this dynamic highlights the urgent need to develop and implement effective rehabilitation programs for individuals suffering from occupationally induced lung damage.

Occupational lung diseases remain a major global public health challenge, particularly in countries with a high burden of mining, metallurgy, and heavy industry. Pneumoconiosis, caused by prolonged inhalation of fibrogenic industrial dusts such as silica, coal, and asbestos, represents one of the most severe and disabling forms of work-related respiratory pathology. Despite advances in occupational safety and exposure control, pneumoconiosis continues to contribute substantially to morbidity, functional disability, and premature loss of work capacity worldwide [5].

The disease is characterized by progressive pulmonary fibrosis, restrictive ventilatory impairment, chronic dyspnea, and reduced exercise tolerance. These physiological changes are frequently accompanied by psychological distress, including anxiety and depressive symptoms, which further aggravate functional limitations and negatively affect quality of life. Importantly, the clinical course of pneumoconiosis is often irreversible, and pharmacological treatment options remain largely supportive rather than disease-modifying [6,7].

In recent decades, exposure patterns have shifted toward complex industrial aerosols, resulting in heterogeneous clinical phenotypes and earlier development of pulmonary hypertension and restrictive lung disorders [8,9]. Delayed diagnosis remains a critical challenge, as early radiological signs of pneumoconiosis are frequently missed due to insufficient screening practices, leading many patients to initiate treatment at advanced and irreversible stages of the disease [10].

Pulmonary rehabilitation has emerged as a cornerstone of care for patients with chronic respiratory diseases. In contrast to pharmacological therapy, non-pharmacological rehabilitation interventions aim to improve functional capacity, alleviate symptoms, and enhance patient-reported outcomes through structured physical training, airway clearance techniques, breathing exercises, occupational therapy, and psychosocial support. While the benefits of pulmonary rehabilitation are well established in chronic obstructive pulmonary disease and interstitial lung diseases, evidence specifically addressing pneumoconiosis remains limited and is often extrapolated from studies including broader interstitial lung disease populations [11,12,13,14,15].

Several national and regional studies have demonstrated that non-pharmacological rehabilitation programs can lead to meaningful improvements in exercise tolerance, respiratory symptoms, and daily functioning among patients with dust-induced occupational lung diseases [16]. These findings suggest that rehabilitation interventions may partially compensate for irreversible structural lung damage by improving physical conditioning, airway clearance, and adaptive coping mechanisms.

Recent clinical investigations report improvements in the six-minute walk distance (6 MWD), reductions in anxiety and depression scores, and clinically significant gains in disease-specific quality-of-life measures following structured rehabilitation programs. Notably, multimodal approaches combining physical training with airway clearance or environmental therapies, such as salt aerosol therapy, appear to yield superior outcomes compared with single-modality interventions [17]. However, the available evidence remains heterogeneous, and the magnitude and consistency of these effects have not yet been comprehensively evaluated.

The aim of this review was to summarize and critically analyze the available evidence regarding pulmonary rehabilitation and related supportive interventions in patients with pneumoconiosis. Despite the growing burden of pneumoconiosis worldwide, evidence on the effectiveness of non-pharmacological rehabilitation interventions remains fragmented and insufficiently synthesized.

## 2. Materials and Methods

### 2.1. Study Design

This systematic review was conducted in accordance with PRISMA (Preferred Reporting Items for Systematic Reviews and Meta-Analyses) 2020 guidelines, where applicable [18]. The study followed a protocol-oriented methodology aimed at identifying, appraising, and synthesizing available evidence on the effectiveness of non-pharmacological rehabilitation interventions in patients with pneumoconiosis and related occupational lung diseases. The review protocol was not prospectively registered. Because the number of studies specifically addressing pulmonary rehabilitation in pneumoconiosis is limited, studies conducted in closely related interstitial lung disease (ILD) populations were considered when they evaluated comparable pulmonary rehabilitation interventions. However, the eligibility criteria, search strategy, and outcomes of interest were defined prior to the literature screening process. Due to the limited number of interventional trials in pneumoconiosis populations, observational and cross-sectional studies were included to contextualize functional and psychosocial outcomes. So, this study was designed as a systematic review of interventional and observational studies.

### 2.2. Research Question and Eligibility Framework

The research question was formulated using the PICO framework (Population, Intervention, Comparison, Outcomes) [19], complemented by the PerSPecTIF model to address the complexity of rehabilitation interventions.

- Population (P): Adult patients (≥18 years) diagnosed with pneumoconiosis, including silicosis, coal workers’ pneumoconiosis, anthracosis, and asbestosis, with or without comorbid chronic obstructive pulmonary disease.
- Intervention (I): Non-pharmacological rehabilitation and spa-based treatment modalities, including pulmonary rehabilitation, physical training, breathing exercises, airway clearance techniques, salt aerosol therapy, physiotherapy, occupational therapy, and specialized nursing care [16].
- Comparison (C): Standard medical therapy, usual care, or control groups without active rehabilitation interventions.
- Outcomes (O): Functional outcomes and patient-reported outcomes assessed using validated instruments, including quality-of-life questionnaires (SGRQ, CAT, SF-36), psychological scales (SAS, SDS), and functional performance tests such as the six-minute walk distance (6MWD).
- Study Design: Randomized controlled trials, prospective studies, cohort studies, and cross-sectional studies.

Eligibility Criteria

Studies were considered eligible if they met the following criteria:

(1) Involved adult patients diagnosed with pneumoconiosis or closely related occupational interstitial lung diseases;
(2) Evaluated pulmonary rehabilitation or related rehabilitation interventions;
(3) Reported clinical, functional, or psychological outcomes;
(4) Were published in peer-reviewed journals in English.

Randomized controlled trials, observational studies, and cross-sectional studies were considered to capture both interventional evidence and descriptive information on rehabilitation needs among pneumoconiosis patients.

### 2.3. Information Sources and Search Strategy

A comprehensive literature search was conducted in international electronic databases, including PubMed/MEDLINE, the Cochrane Library, and Google Scholar. In addition, sources of grey literature were reviewed, including reports and methodological documents from international organizations and regulatory bodies such as the World Health Organization (WHO) and the International Labour Organization (ILO).

The search covered publications from January 2005 to February 2026. Search terms and Medical Subject Headings (MeSH) related to pneumoconiosis, occupational lung diseases, pulmonary rehabilitation, non-pharmacological therapy, and quality of life were combined using Boolean operators. Only studies published in English were considered eligible.

To enhance transparency and reproducibility, the search strategy was developed using a combination of controlled vocabulary terms and free-text keywords related to pneumoconiosis and pulmonary rehabilitation. An example search strategy used for PubMed was as follows: (“pneumoconiosis” OR “coal workers’ pneumoconiosis” OR “silicosis” OR “occupational lung disease”) AND (“pulmonary rehabilitation” OR “respiratory rehabilitation” OR “exercise training” OR “rehabilitation program”). The search strategy was adapted for each database according to its specific indexing system and search interface. Google Scholar was used as a supplementary search tool to identify additional relevant publications and grey literature that may not have been indexed in the primary databases.

### 2.4. Study Selection

Following the removal of duplicate records, titles and abstracts were independently screened by two reviewers according to predefined inclusion and exclusion criteria. Full-text articles were subsequently assessed for eligibility.

Inclusion criteria: The search was limited to studies published from 2005 onwards. This restriction was applied because pulmonary rehabilitation programs and standardized outcome assessment methods became more widely implemented in clinical respiratory research during the mid-2000s.

Exclusion criteria included pediatric populations, conference abstracts without full-text availability, narrative reviews, case reports, and studies lacking statistical analysis or validated outcome measures. Disagreements were resolved through discussion and consensus.

Both interventional and observational studies were considered for inclusion. Interventional studies were primarily used to assess the effectiveness of pulmonary rehabilitation programs, whereas cross-sectional and observational studies were included to provide contextual information on clinical characteristics, rehabilitation needs, and functional limitations among patients with pneumoconiosis. Any disagreements between reviewers were resolved through discussion and consensus.

### 2.5. Data Extraction

Data extraction was also performed independently by two reviewers using a standardized data collection form developed in Microsoft Excel. Extracted variables included author and year of publication, country, study design, sample size, type of rehabilitation intervention, duration of follow-up, outcome measures, and key results. Functional outcomes and patient-reported outcomes were recorded together with effect sizes, confidence intervals, and *p*-values when available.

### 2.6. Risk of Bias and Quality Assessment

The methodological quality of randomized controlled trials was assessed using the Cochrane Risk of Bias 2.0 (RoB 2) tool [20]. Observational studies were evaluated using the Newcastle–Ottawa Scale, while cross-sectional studies were assessed using the AXIS appraisal tool. Quality assessment was performed independently by two reviewers. Overall, the risk of bias across the included randomized controlled trials was assessed, ranging from moderate to acceptable. The main potential sources of bias were the lack of participant blinding and unclear allocation concealment in several trials.

### 2.7. Data Synthesis

Due to substantial heterogeneity in study designs, interventions, and outcome measures, a narrative synthesis approach was applied. The synthesis followed a structured and transparent framework consistent with established guidance for narrative synthesis of heterogeneous evidence.

Specifically, results were organized according to type of intervention and outcome domains (functional capacity, quality of life, and psychological outcomes), allowing for a clinically meaningful interpretation of the findings. In addition, the evidence was stratified by study design (randomized controlled trials, observational studies, and cross-sectional studies) to reflect differences in methodological strength.

Interventional studies were considered the primary source of evidence for evaluating the effectiveness of pulmonary rehabilitation interventions. In contrast, observational and cross-sectional studies were used to provide contextual and supportive evidence, particularly with respect to baseline functional status, psychological burden, and rehabilitation needs among patients with pneumoconiosis.

This structured approach allowed for a consistent comparison of findings across heterogeneous studies while maintaining transparency in the interpretation of evidence.

### 2.8. Ethical Considerations

This study was based exclusively on secondary analysis of published data and did not involve direct interaction with human participants or the use of identifiable personal data. Therefore, approval from an institutional ethics committee was not required.

The review process followed established principles of systematic evidence synthesis to ensure transparency and reproducibility.

## 3. Results

### 3.1. Study Screening and Included Studies

**Figure 1:**
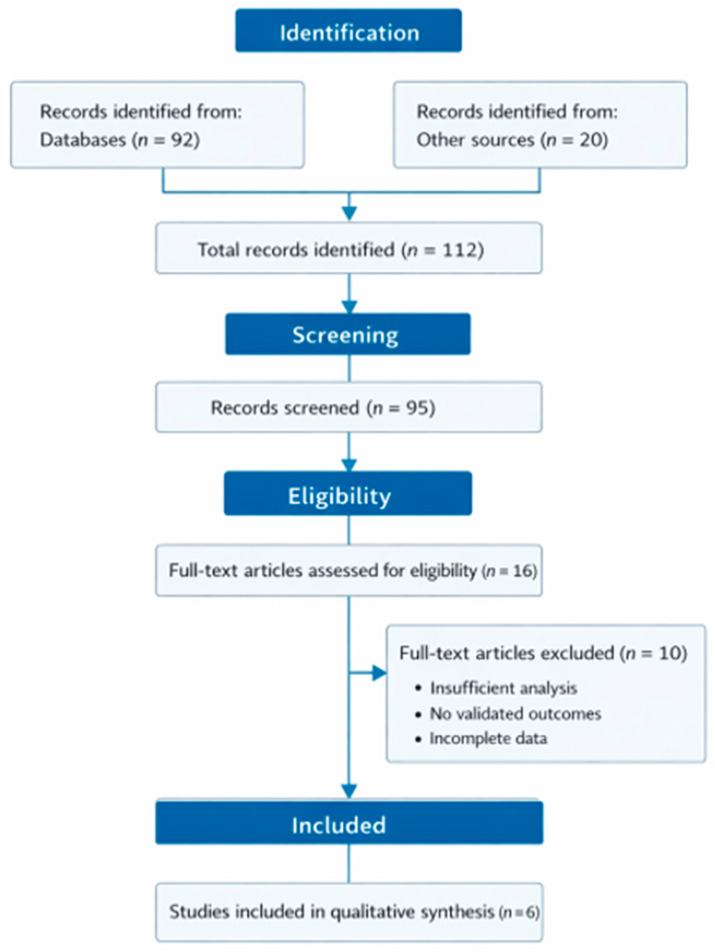
PRISMA 2020 flow diagram of the study selection process.

The systematic search identified a total of 112 records from electronic databases and grey literature sources. After the removal of duplicates, 95 records were screened based on titles and abstracts. Of these, 79 records were excluded. Sixteen full-text articles were assessed for eligibility, of which ten were excluded due to insufficient statistical analysis, absence of validated outcome measures, or incomplete reporting. Ultimately, six studies met the predefined inclusion criteria and were included in the qualitative synthesis. The study selection process is presented in Figure 1 in accordance with the PRISMA 2020 guidelines.

### 3.2. Characteristics of Included Studies

The final analysis comprised 6 studies involving a total of 1049 adult patients diagnosed with pneumoconiosis or related occupational interstitial lung diseases. The included studies were conducted in China, Australia, and Japan. Study designs consisted of randomized controlled trials (RCTs) [21,22] evaluating pneumoconiosis-specific or mixed interstitial lung disease populations with cross-sectional descriptive studies [23], prospective observational studies [24], and cross-sectional studies [25]. Evidence from pulmonary rehabilitation trials in interstitial lung disease was considered when direct pneumoconiosis data were limited [12]. Evidence derived from ILD populations was used cautiously due to limited pneumoconiosis-specific data.

A broad range of non-pharmacological rehabilitation interventions was evaluated, including pulmonary rehabilitation programs, physical training, salt aerosol therapy, acupuncture combined with exercise therapy, occupational therapy, and comprehensive nursing interventions. Outcome measures included disease-specific and generic quality-of-life assessment tools (SGRQ, CAT, SF-12, SF-36, and COPM), psychological assessment scales (SAS, SDS, and MUNSH), and functional performance tests, primarily the six-minute walk distance (6MWD). The results of the risk of bias assessment are summarized in Table 1. 

No studies were excluded on the basis of high risk of bias alone. The main characteristics and outcomes of the included studies are summarized in Table 2.

Given the heterogeneity of study designs, the findings were interpreted according to study type. Interventional studies were used to evaluate the effects of rehabilitation-related interventions on functional outcomes, whereas observational and cross-sectional studies were treated as descriptive evidence and used primarily to provide contextual insights into patient characteristics and rehabilitation needs.

### 3.3. Evidence from Randomized Controlled Trials

Randomized controlled trials provide the strongest evidence regarding the effectiveness of pulmonary rehabilitation interventions in pneumoconiosis and related populations. The included RCTs, such as those conducted by Chen et al. (2022) [21] and Zhang et al. (2016) [22], evaluated structured rehabilitation programs incorporating exercise training, respiratory physiotherapy, and complementary therapeutic approaches. These studies reported improvements in functional capacity, symptom control, and quality-of-life indicators, including improvements in six-minute walk distance and patient-reported outcomes. However, sample sizes were relatively small, and intervention protocols varied across studies.

### 3.4. Evidence from Observational Studies

Observational studies provided additional insights into rehabilitation outcomes among pneumoconiosis patients. For example, Yang et al. (2022) [24] reported improvements in psychological outcomes following comprehensive nursing interventions, including reductions in anxiety scores. However, the absence of randomization and the potential influence of confounding factors limit the strength of causal inference derived from these studies.

### 3.5. Evidence from Cross-Sectional Studies

Cross-sectional studies primarily provided descriptive insights into the clinical characteristics, functional limitations, and psychological burden experienced by pneumoconiosis patients. Studies such as those by Song et al. (2013) [23] and Kawaji et al. (2022) [25] highlighted substantial impairments in quality of life, physical activity, and mental health among affected individuals. While these studies do not evaluate intervention effectiveness, they emphasize the potential need for structured rehabilitation programs in this patient population.

### 3.6. Quality-of-Life Outcomes

Across all included studies, patients with pneumoconiosis demonstrated substantially impaired baseline quality of life. Randomized controlled trials and controlled studies reported statistically significant and clinically meaningful improvements in selected quality-of-life domains following rehabilitation interventions. Dowman et al. [12] observed a reduction of 5.8 points in the SGRQ-I score after exercise-based rehabilitation, exceeding the minimal clinically important difference (MCID) (*p* = 0.04). Notably, this study was conducted in patients with interstitial lung disease rather than pneumoconiosis. Similarly, Chen et al. [21] reported a clinically meaningful reduction in total SGRQ scores following salt aerosol therapy (*p* < 0.05).

Cross-sectional studies confirmed a substantial burden of impaired quality of life among patients with pneumoconiosis. Song et al. [23] reported markedly reduced disease-specific quality-of-life scores in hospitalized patients with coal workers’ pneumoconiosis.

### 3.7. Psychological Outcomes

Psychological distress was a common finding across the included studies. Elevated levels of anxiety and depression were reported in both cross-sectional and prospective investigations. Yang et al. [24] investigated the impact of comprehensive nursing interventions in patients with pneumoconiosis. Their findings indicated improvements in patient-reported outcomes and quality-of-life measures following the intervention. Kawaji et al. [25] identified moderate depressive symptoms, reflected by a mean SDS score of 49, which were significantly associated with dyspnea severity and reduced physical activity levels.

Overall, improvements in psychological outcomes were closely associated with gains in physical functioning and activity performance, suggesting a bidirectional relationship between functional recovery and mental well-being in patients with pneumoconiosis.

### 3.8. Functional Outcomes: Six-Minute Walk Distance

Functional exercise capacity, assessed using the six-minute walk test, improved significantly following rehabilitation interventions, as summarized in Table 3. Across randomized and prospective studies, increases in 6MWD ranged from approximately 25 m in ILD-based rehabilitation trials to 38 m in a pneumoconiosis-specific RCT, exceeding commonly accepted minimal clinically important difference (MCID) thresholds of approximately 25–35 m for chronic respiratory diseases [12,22].

The largest improvement was reported by Chen et al. [21], where salt aerosol therapy resulted in a mean increase of 38 m (95% CI: 28.4–48.3). An improvement in functional exercise capacity was reported following combined acupuncture and exercise therapy; however, the magnitude of change in 6MWD was not quantified [22], while pulmonary rehabilitation programs in interstitial lung disease populations demonstrated an average increase of approximately 25 m [12]. In contrast, likely reflecting their primary focus on activities of daily living rather than aerobic conditioning [24]. Confidence interval analysis indicated greater variability in studies with smaller sample sizes.

### 3.9. Integrated Synthesis of Findings

Across the included studies, generally consistent associations were observed between improvements in functional capacity, enhanced quality of life, and reductions in anxiety and depressive symptoms. Multimodal rehabilitation approaches integrating physical training, airway clearance techniques, and supportive therapies were associated with more consistent improvements across multiple outcome domains for patients with pneumoconiosis.

## 4. Discussion

The available evidence on pulmonary rehabilitation in pneumoconiosis remains limited and heterogeneous. Only three of the included studies directly evaluated rehabilitation-related interventions, highlighting the limited availability of interventional evidence specifically addressing pulmonary rehabilitation in pneumoconiosis patients. Most studies involve small sample sizes and diverse intervention protocols, and only a limited number of randomized controlled trials are available. Therefore, although current findings suggest potential benefits of rehabilitation interventions, the strength of evidence remains relatively low.

The findings of this review should also be interpreted within the broader context of pulmonary rehabilitation research in chronic respiratory diseases. Pulmonary rehabilitation has been widely recognized as an effective supportive intervention for conditions such as chronic obstructive pulmonary disease and interstitial lung diseases, where it has been shown to improve exercise capacity, reduce symptom burden, and enhance quality of life. Although pneumoconiosis differs in etiology and pathophysiology, many functional limitations observed in these patients are comparable to those reported in other chronic respiratory conditions. Therefore, rehabilitation approaches successfully applied in other chronic lung diseases may also have potential relevance for patients with pneumoconiosis, although further disease-specific evidence is required.

The certainty of the available evidence remains limited. Based on the GRADE approach, most of the included evidence would be considered low to very low certainty due to small sample sizes, heterogeneity of interventions, and the predominance of observational study designs.

This systematic review provides a comprehensive synthesis of current evidence regarding the effectiveness of non-pharmacological rehabilitation interventions in patients with pneumoconiosis. The findings demonstrate that structured rehabilitation programs are associated with clinically meaningful improvements in functional capacity, quality of life, and psychological well-being, despite the irreversible nature of dust-induced lung damage. These findings are particularly relevant for respiratory physicians managing occupational lung diseases in industrial and mining regions. Evidence derived from ILD populations should therefore be interpreted cautiously when extrapolated to pneumoconiosis patients. International evidence further supports the feasibility and effectiveness of large-scale rehabilitation systems for patients with pneumoconiosis. China has demonstrated notable success in this area by establishing a nationwide network of pneumoconiosis rehabilitation stations. To date, 829 specialized rehabilitation centers have been implemented, providing more than 1.2 million free rehabilitation services, including treadmill training, breathing exercises, and other supportive interventions. Evidence suggests that such infrastructure-based rehabilitation models not only improve access to care but also contribute to reducing the direct economic burden on affected individuals [26]. The effectiveness of pulmonary rehabilitation at the patient level has also been confirmed by large observational studies. For example, Wang et al. reported outcomes from a cohort of 1994 patients with pneumoconiosis in Chongqing, China, of whom 77.8% participated in structured rehabilitation programs [27]. Comparative analysis revealed significant differences between participants and non-participants with respect to annual hospitalization frequency, hospitalization costs, disability levels, and socioeconomic indicators (*p* < 0.05). These findings highlight that rehabilitation participation is associated not only with clinical benefits but also with broader economic and social impacts, reinforcing the importance of systematic rehabilitation programs in occupational lung disease management.

The heterogeneity observed across the included studies can be explained by several factors. First, the studies involved different types of pneumoconiosis and varying stages of disease severity. Second, pulmonary rehabilitation protocols differed substantially in duration, intensity, and components, including exercise training, education, and breathing techniques. Third, outcome assessment tools varied between studies, particularly for quality-of-life and functional capacity measurements. Finally, follow-up duration differed across studies, which may have influenced the reported sustainability of rehabilitation effects.

It should be noted that some evidence was derived from studies including patients with interstitial lung diseases, which may limit direct extrapolation to pneumoconiosis populations.

One of the most consistent findings across the included studies was the improvement in exercise tolerance, as measured by the six-minute walk distance. Increases ranging from 25 to 38 m exceeded the established minimal clinically important difference for chronic respiratory diseases, indicating not only statistical but also clinical relevance. These results align with previous evidence supporting the benefits of pulmonary rehabilitation in interstitial lung diseases and chronic obstructive pulmonary disease, suggesting that patients with pneumoconiosis may achieve comparable functional gains despite differing pathophysiological mechanisms [12,22,23]. Although improvements in 6MWD exceed commonly cited MCID thresholds derived from chronic respiratory disease populations, it should be noted that only one randomized controlled trial was conducted exclusively in a pneumoconiosis population, while other evidence was derived from mixed or ILD cohorts. Due to the limited number of studies specifically focusing on pulmonary rehabilitation in pneumoconiosis, evidence from studies involving patients with interstitial lung diseases (ILDs) was also considered. However, these findings should be interpreted with caution, as ILD populations may differ in clinical characteristics and disease progression. Therefore, extrapolation of results to pneumoconiosis requires careful consideration.

Quality-of-life outcomes improved across multiple domains, particularly physical functioning and activity limitations. Reductions in SGRQ scores and improvements in CAT and COPM measures highlight the importance of patient-reported outcomes in assessing the effectiveness of rehabilitation interventions. Notably, occupational therapy demonstrated pronounced benefits in activity performance and satisfaction, underscoring the relevance of individualized, goal-oriented rehabilitation approaches for patients with long-standing functional impairment [24]. These findings support a shift toward patient-centered outcome assessment in occupational respiratory medicine. Cross-sectional evidence consistently highlights a profound impairment of quality of life among patients with pneumoconiosis. Interventional studies suggest that selected rehabilitation approaches may partially mitigate this burden, although evidence remains limited.

Psychological distress, including anxiety and depressive symptoms, was prevalent among patients with pneumoconiosis and closely associated with reduced physical capacity and dyspnea severity. Rehabilitation interventions led to significant reductions in anxiety and depressive symptoms, particularly in studies incorporating comprehensive nursing or psychosocial support; however, these findings are primarily based on observational evidence [25,28]. This bidirectional relationship between physical and psychological outcomes highlights the need for integrated rehabilitation models that address both somatic and mental health dimensions.

Multimodal rehabilitation strategies appeared to yield more consistent benefits than single-modality interventions, potentially due to synergistic effects on physical conditioning, symptom control, and psychological adaptation. In a single randomized controlled trial, salt aerosol therapy was associated with clinically relevant improvements in functional capacity and respiratory symptoms in patients with pneumoconiosis [21]; however, evidence from a single randomized trial suggests potential benefit and should be interpreted with caution. These findings suggest that synergistic effects may be achieved through comprehensive rehabilitation programs targeting multiple physiological and psychosocial pathways simultaneously [22,23]. However, heterogeneity in intervention design and outcome measurement precluded quantitative meta-analysis and warrants cautious interpretation.

This review has several limitations. The review protocol was not prospectively registered in PROSPERO. At the time of the study design, the authors developed an internal protocol defining eligibility criteria, outcomes, and search strategy prior to database searching. However, the absence of formal registration may limit transparency, and it represents a limitation of the present review. Then, the number of studies specifically addressing pulmonary rehabilitation in pneumoconiosis remains limited. Furthermore, heterogeneity in study design and outcome measures restricted the possibility of conducting a quantitative meta-analysis. Additionally, some included evidence was derived from studies involving interstitial lung disease populations, which may limit direct generalizability to pneumoconiosis. Finally, long-term follow-up data were scarce, limiting conclusions regarding the sustainability of rehabilitation benefits. In addition, the overall certainty of evidence is limited by the predominance of observational studies and relatively small sample sizes in several included studies. Another limitation of the present review is that only a small number of studies directly evaluated rehabilitation interventions, while several included studies were descriptive in nature and did not assess intervention effectiveness. The heterogeneity of study designs, rehabilitation protocols, and outcome measures limited the possibility of conducting a quantitative meta-analysis.

Despite these limitations, the present review has important strengths. It synthesizes a diverse body of international evidence, incorporates both functional and patient-reported outcomes, and emphasizes clinical relevance through the use of minimal clinically important differences. By focusing specifically on pneumoconiosis, this review addresses a critical gap in the rehabilitation literature and provides a foundation for future research and guideline development. Additionally, the small number of eligible studies limits the generalizability of the findings.

Future development of pneumoconiosis rehabilitation systems should increasingly incorporate digital and remote healthcare solutions. Growing evidence indicates that patients demonstrate substantial demand for “Internet + healthcare” services, particularly at the level of primary rehabilitation stations. The integration of telemedicine and telerehabilitation platforms may reduce geographical and organizational barriers, enhance continuity of care, and improve long-term adherence to rehabilitation programs. International experience with outpatient and telehealth-based pulmonary rehabilitation suggests that such models are feasible and may serve as an effective complement to traditional facility-based approaches, particularly for patients with limited mobility or access to specialized centers [29,30].

From a clinical perspective, the available evidence supports the potential role of non-pharmacological pulmonary rehabilitation as a complementary component of comprehensive management in patients with pneumoconiosis. Structured rehabilitation programs may contribute to improvements in functional capacity and patient-reported outcomes, which are particularly important in chronic occupational lung diseases characterized by progressive functional decline. However, individualized rehabilitation strategies and standardized protocols are needed to optimize clinical effectiveness in this population.

Future research should focus on well-designed randomized controlled trials specifically involving pneumoconiosis populations. Standardized rehabilitation protocols, consistent outcome measures, and longer follow-up periods would help generate stronger evidence regarding the effectiveness of pulmonary rehabilitation in this patient group.

Taken together, the available evidence suggests that pulmonary rehabilitation may represent a promising supportive strategy for patients with pneumoconiosis; however, further high-quality studies are required to confirm these findings.

## 5. Conclusions

The current body of evidence suggests that non-pharmacological pulmonary rehabilitation interventions may provide meaningful benefits for patients with pneumoconiosis, particularly with respect to exercise capacity, health-related quality of life, and psychological well-being. Improvements in functional outcomes, such as the six-minute walk distance, indicate that rehabilitation programs may contribute to clinically relevant improvements in daily functioning.

However, the available evidence remains limited and heterogeneous, and the certainty of the evidence is generally low due to methodological variability, small sample sizes, and the predominance of observational studies. Consequently, the findings of this review should be interpreted with caution.

Further high-quality randomized controlled trials specifically focusing on pneumoconiosis populations are required to confirm the effectiveness of rehabilitation interventions and to establish standardized rehabilitation strategies for patients with occupational dust-related lung diseases.

Nevertheless, pulmonary rehabilitation may represent an important component of comprehensive care for individuals affected by pneumoconiosis. Continued development of accessible and evidence-based rehabilitation programs may contribute to improving long-term functional outcomes and quality of life in this patient population.

## Figures and Tables

**Table 1:** Risk of bias assessment of included studies.

Study	Study Design	Quality Assessment Tool	Selection Bias	Performance Bias	Detection Bias	Overall Risk
Dowman et al. [12] (2017)	Randomized controlled trial	Cochrane RoB 2.0	Low	Moderate	Low	Moderate
Chen et al. [21] (2022)	Randomized controlled trial	Cochrane RoB 2.0	Moderate	Moderate	Moderate	Moderate
Zhang et al. [22] (2016)	Randomized controlled trial	Cochrane RoB 2.0	Moderate	Moderate	Moderate	Moderate
Song ZF et al. [23] (2013)	Cross-sectional study	AXIS appraisal tool	Moderate	—	—	Moderate
Yang et al. [24] (2022)	Prospective observational study	Newcastle–Ottawa Scale	Moderate	—	—	Moderate
Kawaji et al. [25] (2022)	Cross-sectional study	AXIS appraisal tool	Moderate	—	—	Moderate

Note: “—” indicates that the information was not reported in the study.

**Table 2:** Characteristics of the interventional and observational studies included in the systematic review.

Author (Year)	Country	Study Design	Sample Size (*n*)	Study Focus/Intervention	Outcome Measures	Main Findings
Dowman et al. [12] (2017)	Australia	RCT	142	Pulmonary rehabilitation (evidence derived from ILD population)	SGRQ-I, 6MWD	↓ SGRQ by 5.8 points; ↑ 6MWD
Chen et al. [21] (2022)	China	RCT	452	Salt aerosol therapy	SGRQ, 6MWD	↓ SGRQ total score; ↑ 6MWD by 38 m
Zhang et al. [22] (2016)	China	RCT	120	Acupuncture + exercise	CAT, 6MWD	Significant improvement in CAT scores and functional exercise capacity; increase in 6MWD reported, magnitude not quantified.
Song ZF et al. [23] (2013)	China	Cross-sectional descriptive study	88	Quality-of-life assessment (Self-QOL questionnaire)	Self-QOL	Marked impairment of quality of life in hospitalized patients with coal workers’ pneumoconiosis
Yang et al. [24] (2022)	China	Prospective observational study	62	Comprehensive nursing intervention	SAS	Comprehensive nursing intervention was associated with a significant reduction in anxiety levels (SAS score decreased from 58 to 38).
Kawaji et al. [25] (2022)	Japan	Cross-sectional observational	185	Psychological and functional assessment (no rehabilitation intervention)	CAT, SDS	Depressive symptoms were prevalent and significantly associated with dyspnea severity and lower physical activity levels.

Note: The study by Kawaji et al. (2022) [25] was observational and did not include a rehabilitation intervention; it was included to provide contextual information on the psychological and functional characteristics of patients with pneumoconiosis; “↓ indicates a decrease compared to baseline or control; ↑ indicates an increase compared to baseline or control.”

**Table 3:** Effects of rehabilitation interventions on six-minute walk distance (6MWD).

Study	Baseline 6MWD (m)	Post-Intervention 6MWD (m)	Δ6MWD (m)	*p*-Value	Clinical Relevance
Dowman et al. [12] (2017)	✓	✓	≈+25	<0.05	Exceeded MCID
Chen et al. [21] (2022)	✓	✓	+38	<0.01	Exceeded MCID
Zhang et al. [22] (2016)	✓	✓	NR	NR	Qualitative improvement reported (no quantitative 6MWD data)

NR—not reported; ✓ = outcome assessed. The six-minute walk distance (6MWD) was not assessed in studies focusing exclusively on psychological or occupational outcomes.

## Data Availability

Data sharing is not applicable to this article, as no new data were generated or analyzed in this study.

## Abbreviations

The following abbreviations are used in this manuscript:

COPD	Chronic Obstructive Pulmonary Disease
MeSH	Medical Subject Headings
MCID	Minimal Clinically Important Difference
CI	Confidence Interval
CAT	COPD Assessment Test
PRISMA	Preferred Reporting Items for Systematic Reviews and Meta-Analyses
PICO	Population, Intervention, Comparison, Outcome
COPM	Canadian Occupational Performance Measure
SAS	Self-Rating Anxiety Scale
SDS	Self-Rating Depression Scale
MUNSH	Memorial University of Newfoundland Scale of Happiness
SGRQ	St. George’s Respiratory Questionnaire
SF-12	12-Item Short Form Health Survey
SF-36	36-Item Short Form Health Survey
6MWD	Six-Minute Walk Distance
RCT	Randomized clinical trials
RoB	Risk of Bias
